# The Effect of Weaning Practices on the Nutritional and Health Status of Saudi Preschool Children

**DOI:** 10.7759/cureus.47273

**Published:** 2023-10-18

**Authors:** Osman Suliman, Walaa M Alsharif, Emad A Alsaedi, Lama S Alhazmi, Lujain M Reshwan, Noof N Alharbi, Farwa Munir, Amal Surrati

**Affiliations:** 1 Clinical Sciences, Faculty of Medicine, Al-Rayan Colleges, Madinah, SAU; 2 College of Medicine, Faculty of Medicine, Al-Rayan Colleges, Madinah, SAU; 3 College of Medicine, University of Sultan Zainal Abidin, Kuala Terengannu, MYS; 4 Family and Community Medicine, Taibah University, Madinah, SAU

**Keywords:** preschool, health, infants, complementary foods, weaning

## Abstract

Background

Weaning is a complex procedure that gradually introduces complementary foods to the baby's diet. Solid food should be started between the ages of 6 and 12 months. Weaning is a challenging and crucial stage in an infant's development. Extreme caution should be used during weaning an infant because delaying it can cause issues like sluggish growth, difficulties feeding, malnutrition, and iron deficiency.

Objective

The current study aims to determine the impact of delayed or early weaning practices on the nutritional status of preschool children in Saudi Arabia. Data was gathered about the time of complementary food introduction, preferred foods in the initial stages, and a child’s health compared to those practices.

Methodology

By convenient sampling, a cross-sectional study was conducted to gather data from 385 parents of Saudi children at preschool age. Questionnaires were shared online. Data were recorded and analyzed on IBM Corp. Released 2012. IBM SPSS Statistics for Windows, Version 21.0. Armonk, NY: IBM Corp. Descriptive analysis and multivariate ANOVA (MANOVA) tests were performed.

Results

Only 6.23% of the infants were introduced to complimentary food at optimal age (6 to 12 months), whereas 85% were found to have delayed weaning. As per the BMI, 74.4% of preschool children were severely underweight, 53.6% of infants consumed pureed vegetables early during weaning, and 64% of infants were introduced to eggs and cheese within the first year of life. The timing, pattern, and food items of weaning had a significant (p<0.05) impact on general physical health, as 48.8% of children had pale skin, 46.9% felt tired, 36.5% had swollen joints, and 42% complained of itching and an upset stomach.

Conclusion

This study couldn’t define the direction of significance. Further studies can be done on a larger scale where biochemical tests, and screening can be done on children to find if any significant health problem is prevailing, and the direction of association can be defined.

## Introduction

Weaning practices mean introducing semi-solid or solid food into an infant’s diet, breastfeeding, or formula milk. When solid food is introduced, providing babies with the proper nutrition during infancy is critical for optimum growth and development [1]. The World Health Organization (WHO) and the American Academy of Pediatrics advocate promoting nursing exclusively for the first six months of a baby's life, with continuous breastfeeding up to 12 months of age or longer, along with the introduction of solid meals [2].

Malnutrition in childhood can refer to both undernutrition and overnutrition. High protein diet intake in infancy can lead to childhood obesity, specifically in the case of the amino acid leucine, which acts as an insulin-like growth factor and increases the chances of weight gain in the early years [3]. Some European nations appear to have reached a plateau in the trend towards rising childhood obesity prevalence rates. Given that early newborns rely on adults for nutrition, parental attitudes, beliefs, and actual feeding habits directly affect the nutritional condition of their offspring.

One of the best-known measures of economic development is the nutritional status of children. The UN endorsed one of the eight Millennium Development Goals emphasized in 2000. Growth indices, such as weight for height, age, and period, are crucial instruments for determining a child's nutritional condition [4].

Current scientific studies exploring the causes of childhood obesity have discovered links between the supplementary feeding practices of parents and the likelihood of childhood obesity. Family mealtimes, awareness of baby hunger, appropriate reactions to satiety cues, and parental feeding habits may all influence the risk of obesity [5].

Along with the dietary sources, the pattern of feeding and the time of solid food introduction also matter. Early introduction of complementary foods can also be unhealthy for infants. During weaning an infant, extreme caution should be used because delaying it can cause issues like delayed growth, difficulty in feeding, malnutrition, and iron deficiency [6].

The advantages of breastfeeding for both the mother and the child are well known, and the health risks of infant formula feeding are becoming better known. Breastfeeding shields infants from infectious diseases during infancy. Breastfeeding throughout infancy offers protection from contagious diseases and is linked to long-term advantages in several domains, including lowered risk of cardiovascular disease, increased intelligence, and reduced allergy symptoms [7]. Breastfeeding has a significant positive impact on a child's health, even in developed nations. However, after a certain age, breastfeeding is insufficient for the baby’s needs as it does not provide sufficient nutrients as required by a growing infant. According to the WHO (2015), human milk is vital to a child's overall health and wellness and can meet one-third of a child's energy demands between 12 and 24 months [8].

Weaning methods impact early childhood eating choices and health. The findings of a study imply that babies weaned using the baby-led method develop the ability to control their food intake, which results in a reduced BMI and a preference for nutritious foods like carbohydrates. The fight against the well-documented growth of obesity in modern society could be affected by this [9].

The introduction of complementary foods is one of the crucial parts. As per a study conducted in Bangladesh, delays in weaning practices can be because of the busy domestic schedules of mothers. As well, mothers limit supplemental feeding because they think some foods cause "stomach problems" and because it takes longer [10]. Improvements to complementary feeding practices have been made using a variety of tactics. They include providing complementary foods, nutritional advice to moms to encourage healthy feeding habits, and supplementing with foods enriched with numerous micronutrients or more energy [11].

In Saudi Arabia, studies that directly focus on the weaning practices implied by Saudi mothers and their impact on later childhood have yet to be conducted. So, this study is planned to find the pattern of weaning practices of Saudi infants and its impact on their physical health outcomes in childhood. The results of the current study will help fill the gap in the literature.

## Materials and methods

A cross-sectional study was conducted where the data was collected from Saudi natives of Saudi Arabia residing in different cities. A convenient non-probability sampling technique was used in the current research. Data was collected and analyzed for the present study from July 2023 until October 2023. The study population was children, both girls and boys, before preschool, living in Saudi Arabia based on inclusion and exclusion criteria. The inclusion criteria set was that Saudi residents before the preschool age who have no congenital or major health problem and Saudi residents aged more than six years who have any congenital or major health problem were excluded. Data was collected from their mothers. In Saudi Arabia, 5.67 million children aged 0-9 years [12] According to the current study, taking a 95% confidence interval, the sample size of the present study was 385 samples. Raosoft, a web-based software, was used to calculate the study's sample size.

A questionnaire was developed to obtain relevant information and was divided into four sections: In Section I, the demographic data of children was taken. Section II asked about clinical symptoms that can help define a child’s health [13]. In Section III, six questions were asked about introducing complementary foods, time, and patterns of weaning practices [14]. In the last section, food options were given to ask which foods were presented to infants within the first year of life [15]. 

Data obtained from this study was analyzed using IBM Corp. Released 2012. IBM SPSS Statistics for Windows, Version 21.0. Armonk, NY: IBM Corp. A value of 0.05 was considered the statistical significance value for all statistical tests in the present study.

Ethical approval for conducting the study was obtained from the Al-Rayan Research Ethics Committee (registered with the National Bioethics Committee in KACST, Saudi Arabia). The study ID was HA-03-M-122-049, dated July 19, 2023. Online consent from the participants was obtained. Questions were asked, and approval was taken in Arabic. They were independent in deciding whether they wanted to fill out the form. The personal data of participants was kept confidential. 

## Results

Distribution of samples based on the introduction of weaning practices

**Figure 1:**
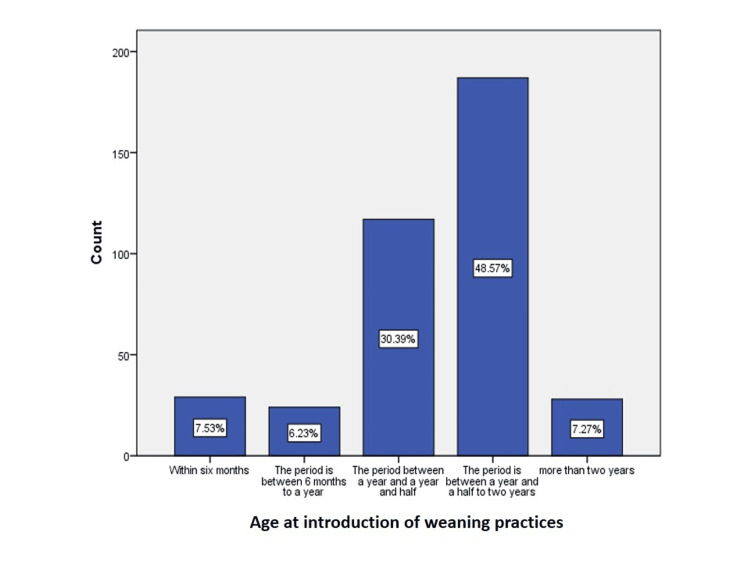
Age distribution as per the introduction of weaning practices The data has been represented as the percentage of occurrence of the variables

A total sample of 385 children was taken under study. The retrospective data was collected based on the mother’s perspective. Mothers were asked about the age when they first introduced any complementary food to their children besides milk. According to the analysis, it was found that 48.57% of mothers introduced weaning practices between the ages of one and a half and two years, 30.39% introduced it at the age of one and a half years, and 7.53% introduced it within six months. 7.27% did it after two years, and 6.23% introduced it between six months and a year. The data is illustrated in Figure 1.

Descriptive analysis of demographic data

Samples were divided based on their age of weaning, and their demographic data were analyzed. Out of a total, 56.7% were males, 35.8% were of age 1-3 years, primarily children were severely underweight, 74.4%, and 84.4 million were not even registered in schools. The data is summarized in Table 1.

**Table 1:** Demographic data distribution based on the age of weaning introduction The data has been represented as the frequency and percentage of occurrence of the variables

	≤6 months	6-12 months	12-18 months	18-24 months	≥24 months	Total
Gender						
Male	17 (58.4%)	13 (54.2%)	59 (50.4%)	111 (59.4%)	19 (67.9%)	219 (56.7%)
Female	12 (41.4%)	11 (45.8%)	58 (49.6%)	76 (40.6%)	9 (32.1%)	167 (43.3%)
Age						
0-1 year	2 (6.9%)	6(25%)	23 (19.7%)	63 (33.7%)	11 (39.3%)	105 (27.2%)
1-3 year	13 (44.8%)	4 (16.7%)	54 (45.2%)	61 (32.6%)	6 (21.4%)	138 (35.8%)
3-5 year	5 (17.2%)	6 (25%)	31 (26.5%)	52 (27.8%)	6 (21.4%)	100 (25.9%)
5-7 year	9 (31.0%)	8 (33.3%)	9 (7.7%)	11 (5.9%)	5 (17.9%)	43 (11.1%)
BMI (lb/inches^2^)						
>17.4 (severely underweight)	15(68.2%)	11(45.8%)	88 (75.9%)	156 (84.8%)	17 (68%)	287(74.4%)
17.5-18.4 (underweight)	0	2 (8.3%)	3 (2.6%)	3 (1.6%)	4 (16%)	12 (3.1%)
18.5-25 (normal)	4(18.2%)	2 (8.3%)	8 (6.9%)	5 (2.7%)	1(4%)	20 (5.2%)
25.1-30 (overweight)	2(9.1%)	1 (4.2%)	3 (2.6%)	5 (2.7%)	0	9 (2.3%)
30.1-40 (0bese)	1(4.5%)	3 (12.5%)	5 (4.3%)	9 (4.9%)	1 (4%)	20 (5.2%)
>40.1 (severely obese)	0	2 (8.3%)	9 (7.8%)	6 (3.3%)	2 (8%)	20 (5.2%)
School Grade						
Not registered	18 (62.1%)	16 (66.7%)	102 (87.2%)	168 (89.8%)	21 (75%)	325 (84.2%)
Primary grades	6 (20.7%)	5 (20.8%)	9 (7.7%)	11 (5.9%)	2 (7.1%)	33 (8.5%)
Elementary	5 (17.2%)	3 (12.5%)	6 (5.1%)	8 (4.3%)	5 (17.9%)	27 (7%)

Practices before and after the introduction of weaning 

Further, questions were asked about the type of milk given before weaning. 57.1% of the infants were given formula milk, and none of the mothers had been given cow milk before the introduction of weaning. Out of the total, 71.4% of the mothers didn’t even introduce cow milk in the first year of life. 50% of the mothers introduced solid food after six months, and 42.9% did it in 4-6 months of infant age. As per the initial solid food, 53.9% of mothers preferred pureed vegetables for their infant, and 32.1% chose the serving frequency once a day. The recorded responses represent that more than half of the mothers had healthier and right practices related to complementary food introduction in their infant diet. The data is summarized in Table 2.

**Table 2:** Practices before and after the introduction of weaning The data has been represented as the frequency and percentage of occurrence of the variables

	≤6 months	6-12 months	12-18 months	18-24 months	≥24 months
Milk before weaning					
Breast milk	15 (51.7%)	9 (37.5%)	94 (80.3%)	159 (85%)	7 (25%)
Formula milk	8 (27.6%)	4 (16.7%)	9 (7.7%)	19 (102%)	16 (57.1%)
Cow milk	0	0	0	0	0
Both (breast and formula milk)	6 (20.7%)	11 (45.8%)	14 (12%)	9 (4.8%)	5 (17.9%)
Cow milk introduction in the first month					
Yes	10(34.5%)	8 (33.3%)	10 (8.5%)	7 (3.7%)	8 (28.6%)
No	19 (65.5%)	16 (66.7%)	107 (91.5%)	180 (96.3%)	20 (71.4%)
Introduction of solid food					
Less than four months	9 (31%)	1 (4.2%)	3 (2.6%)	4 (2.1%)	2 (7.1%)
4-6 months	4 (13.8%)	7 (29.2%)	20 (17.1%)	25 (13.4%)	12 (42.9%)
More than six months	16 (55.2%)	16 (66.7%)	94 (80.3%)	158 (84.5%)	14 (50%)
Type of Food					
Rice and cereal	6 (20.7%)	5 (20.8%)	6 (5.1%)	9 (4.8%)	9 (32.1%)
Pureed Vegetables	15 (51.7%)	13 (54.2%)	70 (59.8%)	96 (51.3%)	15 (53.6%)
Pureed fruits	8 (27.6%)	6 (25%0	41 (35%)	82 (43.9%)	4 (14.3%)
Frequency of solid food					
Didn’t give	5 (17.2%)	2 (8.3%)	9 (7.7%)	21 (11.2%)	6 (21.4%)
Once a day	7 (24.1%)	13 (54.2%)	31 (26.5%)	37 (19.8%)	9 (32.1%)
Two to three times	9 (31%)	5 (20.8%)	49 (41.9%)	97 (51.9%)	7 (25%)
More than three times	3 (10.3%)	0	2 (1.7%)	5 (2.7%)	3 (10.7%)
Once in this period	5 (17.2%)	4 (16.7%)	26 (22.2%)	27 (14.4%)	3 (10.7%)

Food items introduced within the first year of life

**Figure 2:**
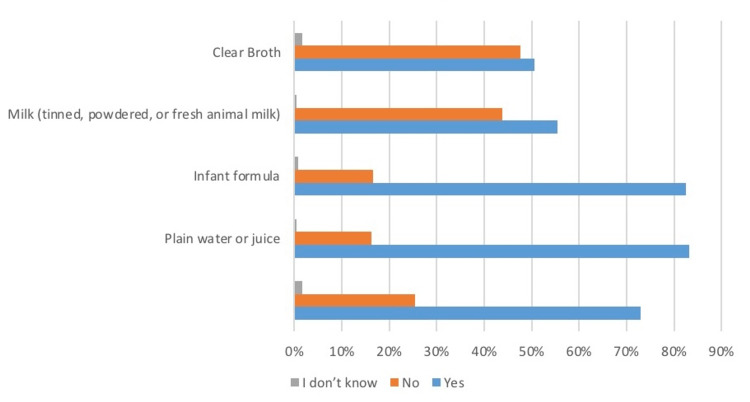
The liquid intake pattern of infant before six month of age The data has been represented as a percentage occurrence of the variable

**Figure 3:**
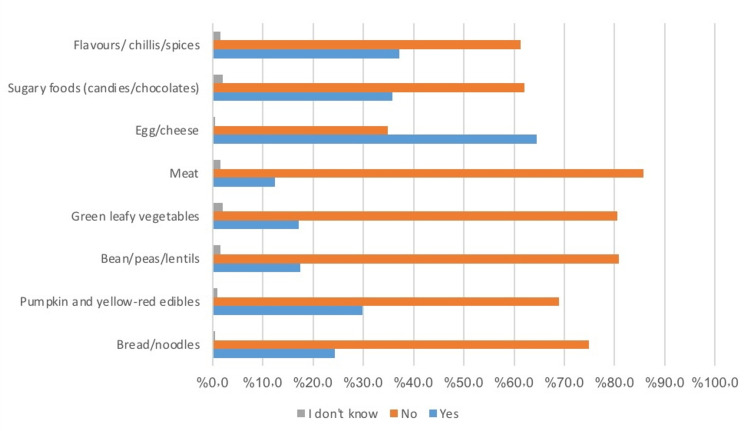
The solid intake pattern of infant between the age of 6-12 months The data has been represented as a percentage occurrence of the variable

In the third section (Table 3), mothers were asked about the food items they introduced in the first six months and then from the sixth month to one year. "As per the given options, 73% of mothers gave their infants ORS or vitamins in the first six months ”, 83.1% gave water or juices, 82.4% gave milk, and 50.55 gave clear broth to their infants. Based on the food items introduced in infants aged six months to a year, only 24.4% of mothers gave bread or noodles, 29.9% gave pumpkin or any yellow or red edible, only 17.4% introduced beans and lentils, and 17.1% gave green leafy vegetables. 12.4% gave meat, and 64.5% of mothers had given eggs and cheese as solid foods in 6-12 months. 35.8% gave sugary foods, and 37.1% introduced chili and flavors in the food of their infants. Data are summarized in Figures 2, 3. 

**Table 3:** Food items introduced within the first year of life The data has been represented as the frequency and percentage of occurrence of the variables

Did you give any of the following liquids to your infants before six months of age			
	Yes	No	I don’t know
Vitamin or ORS	281 (73%)	98(25.5%)	6(1.6%)
Plain water or juice	320 (83.1%)	63 (16.3%)	2 (0.5%)
Infant formula	318(82.4%)	64 (16.6%)	3 (0.8%)
Milk (tinned, powdered, or fresh animal milk)	214 (55.4%)	169(43.8%)	2(0.5%)
Clear Broth	195 (50.5%)	184(47.7%)	6 (1.6%)
Did you give your infant any of the following solids from age 6-12 months?			
	Yes	No	I don’t know
Bread/noodles	94 (24.4%)	289 (74.9%)	2 (0.5%)
Pumpkin and yellow-red edibles	115 (29.9%)	266 (68.9%)	4 (1%)
Bean/peas/lentils	67 (17.4%)	312 (80.8%)	6 (1.6%)
Green leafy vegetables	66 (17.1%)	311 (80.6%)	8 (2.1%)
Meat	48 (12.4%)	331 (85.8%)	6 (1.6%)
Egg/cheese	249 (64.5%)	134 (34.8%)	2 (0.5%)
Sugary foods (candies/chocolates)	138 (35.8%)	239(62.1%)	8(2.1%)
Flavours/ chillis/spices	143 (37.1%)	236 (61.3%)	6 (1.6%)

Distribution of samples as per their health issues

**Figure 4:**
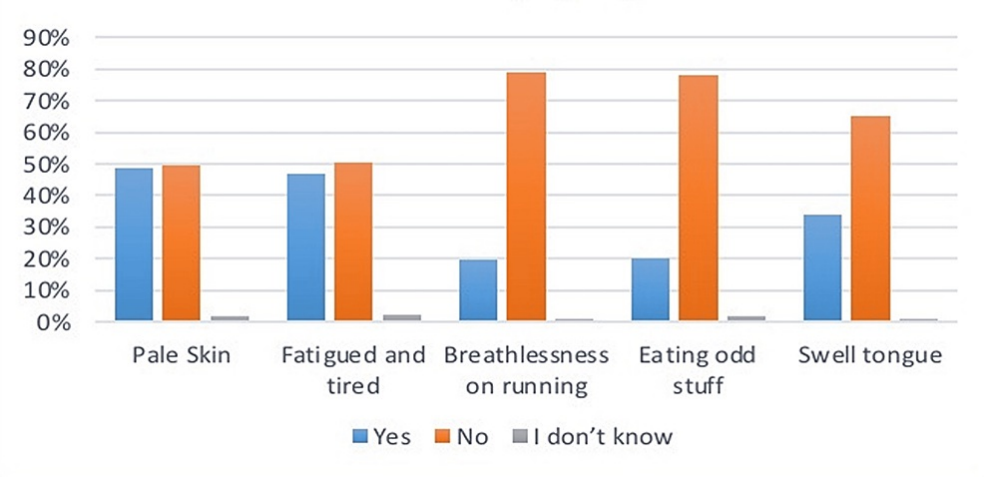
Prevalence of iron deficiency symptoms in infants The data has been represented as a percentage occurrence of the variable

**Figure 5:**
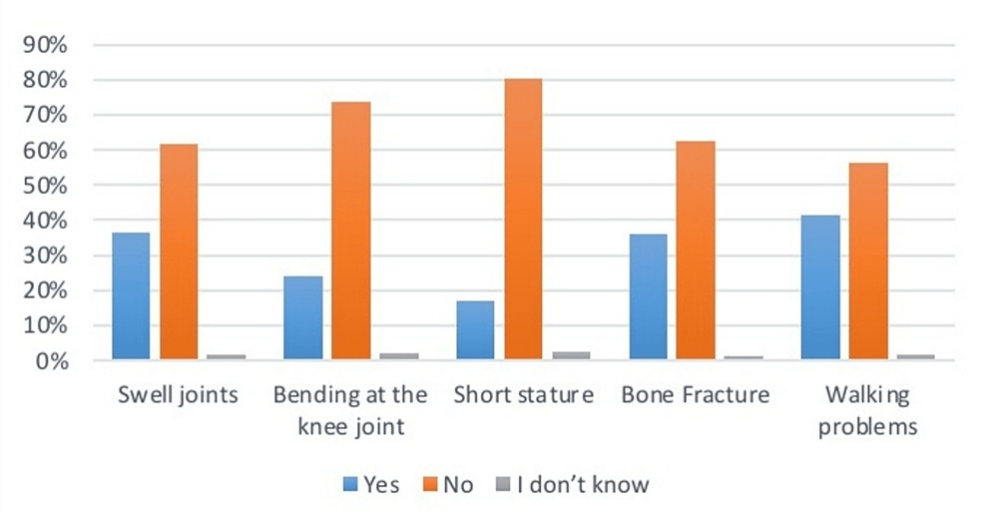
Prevalence of musculoskeletal issues in infants The data has been represented as a percentage occurrence of the variable

**Figure 6:**
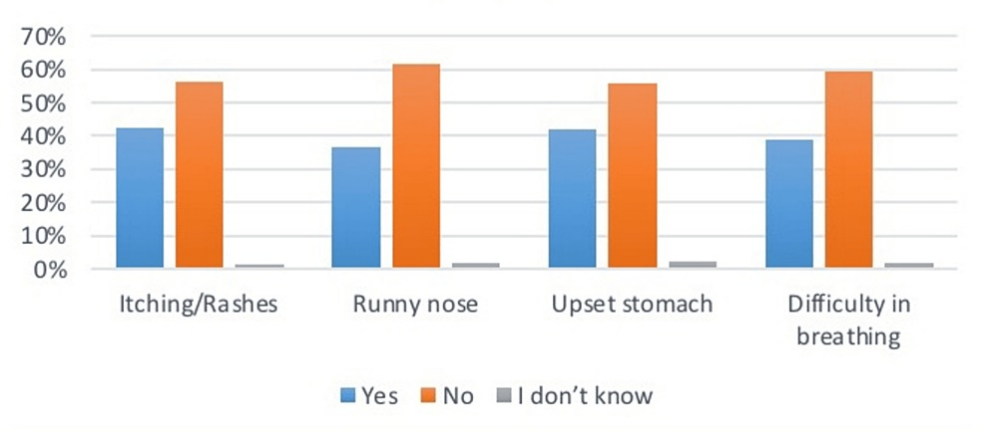
Prevalence of allergic symptoms in infants The data has been represented as a percentage occurrence of the variable

Upon asking questions related to the health problems (Table 4) faced by the children. Mothers responded that 48.8% of children had pale skin, 46.9% felt fatigued, 19.7% became breathless on running, 20.3% ate odd stuff such as dirt, and 33.8% had swollen tongues. As per the musculoskeletal issues, 36.8% had swell joints, 24.2% had bending legs at the point of knees, 17.1% had short stature, and 36% had faced bone fractures. Related to the allergy symptoms, 42.5% complained of itching, 36.6% had the problem of runny nose, 42.1% had upset stomach, and 38.9% had the issue of difficulty in breathing. From the results, it was seen that allergic symptoms were the most common among all health problems recorded in children. Data is summarized in Figures 4-6.

**Table 4:** Health issues in children under study The data has been represented as the frequency and percentage of occurrence of the variables

Health issue	Frequency (%)		
	Yes	No	I don’t know
Iron deficiency symptoms			
Pale Skin	188 (48.8%)	190 (49.4%)	7 (1.8%)
Fatigued and tired	181 (46.9%)	195 (50.5%)	9 (2.3%)
Breathlessness on running	76 (19.7%)	305 (79%)	4 (1%)
Eating odd stuff	78 (20.3%)	301 (78%)	6 (1.6%)
Swell tongue	130 (33.8%)	252 (65.3%)	3 (0.8%)
Musculoskeletal issues			
Swell joints	141 (36.5%)	237 (61.6%)	7 (1.8%)
Bending at the knee joint	93 (24.2%)	284 (73.8%)	8 (2.1%)
Short stature	66 (17.1%)	309 (80.3%)	10 (2.6%)
Bone Fracture	139 (36%)	241 (62.4%)	5 (1.3%)
Walking problems	160 (41.5%)	218 (56.5%)	7 (1.8%)
Allergy Symptoms			
Itching/Rashes	164 (42.5%)	216 (56.1%)	5 (1.3%)
Runny nose	141 (36.6%)	238 (61.8%)	6 (1.6%)
Upset stomach	162 (42.1%)	215(55.7%)	8(2.1%)
Difficulty in breathing	150 (38.9%)	229 (59.3%)	6 (1.6%)

Introduction of food items within six months and their impact on health

To find the impact of weaning practices on children’s health, a multivariate ANOVA (MANOVA) test was performed. The results suggest a significant association (p<0.05) between the type of milk given before weaning and fatigue and a swollen tongue. The introduction of vitamins, water, juice, infant formula, and broth is also significantly (p<0.05) associated with health problems. The data is summarized in Table 5.

**Table 5:** Impact of food items introduced within six months on health The data has been represented as per the p-values of correlation among variables

Source	Dependent Variable	Degrees of Freedom	Mean Square	F	Sig.
Age of weaning introduction	Upset_stomach	4	1.018	4.822	.001
What kind of milk did you give your baby from birth until weaning?	Fatigued_tired	2	1.188	7.068	.001
	Swollen_tongue	2	.529	3.213	.041
	Swell_joints	2	1.415	7.696	.001
	Walking_problem	2	1.382	7.320	.001
	Runny_nose	2	.818	4.028	.019
	Difficulty_in_breathnig	2	.895	5.223	.006
Vitamin drops or other medicines such as drops or ORS	Pale_skin	1	6.561	35.628	.000
	Fatigued_tired	1	5.611	33.398	.000
	Swollen_tongue	1	2.363	14.355	.000
	Swell_joints	1	1.387	7.544	.006
	Walking_problem	1	2.654	14.057	.000
	Rash_itching	1	3.794	17.884	.000
	Runny_nose	1	2.045	10.066	.002
	Difficulty_in_breathnig	1	2.859	16.679	.000
Plain water, Juice or juice drinks	Fatigued_tired	1	.705	4.195	.041
	Walking_problem	1	1.104	5.848	.016
Infant formula (add locally available brand names of infant formula)	Breathless_on_running	1	.985	6.058	.014
	Swell_joints	1	2.689	14.624	.000
	Rash_itching	1	2.102	9.907	.002
	Difficulty_in_breathnig	1	1.208	7.044	.008
Clear broth	Eat_dirt_odd_stuff	1	1.615	9.439	.002
	Pale_skin	1	1.735	9.424	.002
	Fatigued_tired	1	2.624	15.621	.000
	Swollen_tongue	1	2.631	15.985	.000
	Bending_at_knee_joint	1	.950	4.914	.027
	Bone_fracture	1	2.401	12.523	.000
	Walking_problem	1	1.998	10.579	.001
	Rash_itching	1	1.689	7.958	.005
	Difficulty_in_breathnig	1	1.225	7.144	.008

Introduction of food items from six months to one year and their impact on health

The multivariate ANOVA has shown that introducing food items such as bread, rice, yellow-orange vegetables and fruits, beans, meat, eggs, sugary foods, and flavors within a year of age significantly affects health problems (p<0.05). In contrast, the direction of the association cannot be determined (Table 6).

**Table 6:** Impact of food items introduced within one year on health The data has been represented as per the p-values of correlation among variables

Source	Dependent Variable	Degrees of Freedom	Mean Square	F	Sig.
Bread, rice, noodles, or other foods made from grains, including thick grain-based porridge?	Difficulty_in_breathnig	1	2.241	11.986	.001
	Short_strature	1	.702	4.071	.044
	Bending_at_knee_joint	1	1.691	8.450	.004
	Swell_joints	1	.850	4.368	.037
	Fatigued_tired	1	.796	4.052	.045
	Breathless_on_running	1	1.603	10.271	.001
Pumpkin, carrots, squash, or sweet potatoes, Ripe mangoes, ripe papayas that are yellow or orange inside?	Rash_itching	1	3.813	16.622	.000
	Runny_nose	1	4.124	18.392	.000
	Upset_stomach	1	6.879	31.182	.000
	Difficulty_in_breathnig	1	6.781	36.262	.000
	Walking_problem	1	5.415	26.619	.000
	Bone_fracture	1	5.339	27.345	.000
	Short_strature	1	.670	3.885	.049
	Swell_joints	1	6.467	33.240	.000
	Pale_skin	1	6.803	33.551	.000
	Fatigued_tired	1	7.150	36.413	.000
	Breathless_on_running	1	1.473	9.442	.002
	Swollen_tongue	1	6.025	33.791	.000
Any foods made from beans, peas, lentils or nuts, including Plumpy ‘nut?	Walking_problem	1	1.630	8.015	.005
Eggs, Cheese, yoghurt, or other milk products?	Rash_itching	1	3.935	17.152	.000
	Runny_nose	1	3.883	17.317	.000
	Upset_stomach	1	4.931	22.350	.000
	Difficulty_in_breathnig	1	6.267	33.513	.000
	Walking_problem	1	3.455	16.984	.000
	Bone_fracture	1	3.508	17.966	.000
	Bending_at_knee_joint	1	1.915	9.568	.002
	Swell_joints	1	2.366	12.162	.001
	Pale_skin	1	2.403	11.853	.001
	Fatigued_tired	1	5.601	28.522	.000
	Breathless_on_running	1	4.182	26.799	.000
	Swollen_tongue	1	1.944	10.901	.001
Any sugary foods such as chocolates, sweets, candies, pastries, cakes or biscuits?	Difficulty_in_breathnig	1	1.078	5.762	.017
	Walking_problem	1	3.105	15.265	.000
	Bone_fracture	1	1.071	5.487	.020
	Swell_joints	1	2.519	12.945	.000
	Pale_skin	1	6.401	31.569	.000
	Fatigued_tired	1	3.576	18.211	.000
	Breathless_on_running	1	.923	5.916	.015
	Swollen_tongue	1	2.360	13.234	.000
Condiments for flavour, such as chillies, spices, herbs or fish powder?	Rash_itching	1	2.371	10.337	.001
	Upset_stomach	1	1.827	8.279	.004
	Walking_problem	1	1.182	5.810	.016
	Bone_fracture	1	1.583	8.105	.005
	Swell_joints	1	1.598	8.213	.004
	Fatigued_tired	1	1.924	9.796	.002
	Swollen_tongue	1	.903	5.063	.025
Any dark green leafy vegetables?	Walking_problem	1	.784	3.852	.050
	Short_strature	1	1.061	6.155	.014
	Swell_joints	1	.887	4.560	.033
Any meat such as beef, fish, lamb, goat, chicken, duck, or other organ meats?	Pale_skin	1	1.194	5.888	.016
	Fatigued_tired	1	.805	4.101	.044

## Discussion

Every child has the right to optimal emotional, social, and cognitive growth. Diet in the early years of life significantly affects one’s life. Considering the nervous system, the brain's mental, social, and emotional functions continue to grow throughout the lifespan. The CNS development trajectory varies over time, though. Before age three, the brain begins to shape most of its final structure and capacity. Failure to maximize brain development early in infancy appears to have long-term effects on education, employment potential, and adult mental health [16]. Such health issues are also seen in other body systems. 

Great emphasis has been placed on exclusive feeding and lactation in the early months of infancy. The careful introduction of complementary foods is also a crucial factor to consider. This study was planned to investigate the timing and pattern of weaning practices in Saudi infants and their role in their health in the late years.

The American Academy of Pediatrics and the World Health Organization advise exclusively breastfeeding infants for the first six months of life (World Health Organization, 2002). As per the data and concepts of breastfeeding, two methods are generally accepted: to breastfeed exclusively until four months of age and to introduce complementary foods such as a liquid and semi-solid diet. The other concept is to introduce complementary foods after six months of age. As per one of the systemic reviews of literature, it has been concluded that, compared to infants who began receiving mixed breast milk at 3 or 4 months, infants who are exclusively breastfed for six months suffer from gastrointestinal tract infections at a lower morbidity rate. When infants are exclusively breastfed for six months or longer, neither those from developing nations nor those from developed nations show any growth deficits [17]. As per the current study findings, only 6.23% of mothers introduced weaning at the optimal age of their infant; otherwise, 86.24% delayed the introduction of complementary foods.

**Figure 7:**
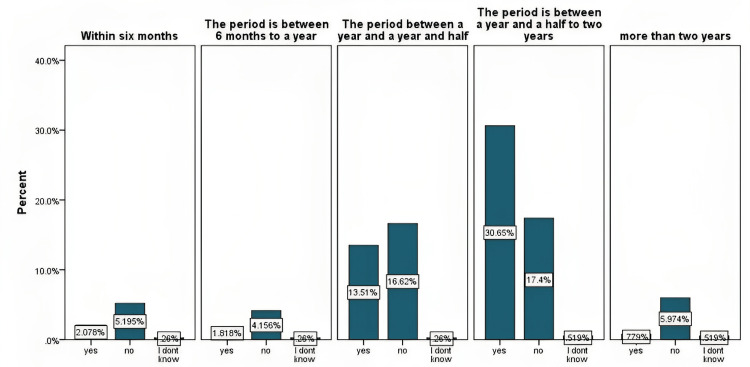
Age of weaning introduction and the onset of pale skin The data has been represented as the percentage of occurrence of the variables

It is ultimately the best time to introduce new foods to an infant after six months, but if mothers raise them later, it can cause multiple health deficits. Many motor and neural development issues are recorded in children with late weaning. As iron deficiency anemia is prevalent among children, it has been found that late weaning is one of its most significant contributors [18]. Looking at the stats of the current study, only 6.23% of mothers introduced weaning between six months to one year, 30.39% introduced it between the ages of 12 to 18 months, 48.57% did it between 18 months to 24 months, and 7.27% did it even after 24 months. These stats show that most Saudi mothers prefer and practice late weaning in their infants. Questions related to anemia symptoms, such as pale skin, breathlessness, fatigue and swollen tongue, were asked. The data from the current study suggest that these symptoms were more common among the children who had the complimentary food introduction between the ages of 18 months to 24 months, supporting the literature. Figure 7 illustrates the result for the question regarding the pale skin of children. 

Traditional weaning foods are typically non-dairy family dishes based on a regional staple, typically a cereal like corn, sorghum, millet, or rice. Sometimes, non-cereal basics like potatoes, cassava, and plantains are used. A staple is generally served as a weaning food as either a thick porridge or a soft or watery gruel. These foods will become voluminous when prepared as liquids because they can hold a lot of water, which results in a low nutritional and energy density [19]. So, frequency and volume of food are also essential in weaning practices. In the current study, mothers were asked about the frequency of food in a day; 32% gave once a day, 25% gave two to three times, and 10% gave complementary food to their infants more than three times a day. 

Several studies have previously investigated the role of complementary foods and food allergies. Food allergies have been commonly found in Western nations. Foods containing allergens are suggested to be added to the diet after one year of age. It can be wheat, cow milk, peanuts, or proteins. Studies have concluded that allergic reactions are not confined to food items alone. Many factors are yet to be linked [19]. This survey asked about the food items introduced from six months to one year. It was concluded that 64.5% of mothers introduced eggs and cheese to their infants, 35.5% introduced sugar foods, 37% added flavors and spices, and 29.9% added pumpkin and yellow vegetables to their diet. In contrast, as per the allergic symptoms, 42.5% complained of rashes and itching, 36.6% complained of a runny nose, 42.1% complained of an upset stomach, and 38.9% complained of difficulty breathing. A significant association was also noticed between symptoms and food item consumption, but this study couldn’t define the direction of the association. 

Breast milk does not provide enough calories, proteins, zinc, iron, or fat-soluble vitamins (vitamins A, D, and K) to ensure the infant's growth throughout the second half of the first year of life. So, adding essential vitamins and proteins is one of the primary needs for an infant [20]-meat poultry. Cheese, dairy, and yellow and red food items such as pumpkin, carrot, mangoes, and papaya are famous sources of fat-soluble vitamins and protein, and it has been seen in the current study that most mothers introduced such food items in their infant diet after six months. Vitamin K and D deficiencies are associated with skeletal systems, so the symptoms were analyzed, and it was seen that 24% of the children had complaints of bending legs. A significant association was found between food consumption and skeletal issues, but the direction of significance was not defined. 

There were a few limitations to the current study. First, the data was collected online. Face-to-face data collection can ensure that the participant has rightly understood the question. Second, the study collected retrospective data, which can result in missing data or personal bias among the participants.

## Conclusions

A significant association was found among the weaning practices, patterns, and food items with the health issues highlighting iron deficiency, musculoskeletal problems, and allergies. In contrast, the current study could not define whether this association negatively or positively impacted health. It was a retrospective, cross-sectional study. To get better results, a prospective longitudinal study can be done on a similar topic to get more precise results. This study was unable to define the direction of the association, so studies should be conducted to determine if the introduction of certain food items to infants has a negative impact.
